# Psychometric evaluation of the Swedish Cognitive Fusion Questionnaire-7: a brief report

**DOI:** 10.3389/fpsyg.2026.1922745

**Published:** 2026-07-30

**Authors:** Andreas Larsson

**Affiliations:** Department of Pedagogy, Psychology, and Social Work, Mittuniversitetet - Campus Ostersund, Östersund, Sweden

**Keywords:** CFQ-7, cognitive fusion, psychological flexibility, Rasch model, validation

## Abstract

**Background:**

The Cognitive Fusion Questionnaire-7 (CFQ-7) assesses cognitive fusion, a core psychological flexibility process, but Swedish item-level evidence remains limited.

**Objectives:**

This brief report evaluated reliability, internal structure, response-category functioning, and construct validity of the Swedish CFQ-7 in an adult Swedish-language sample.

**Methods:**

Secondary analyses used data from 432 Swedish adults; 429 had complete CFQ-7 item data. Analyses included reliability, ordinal CFA, Rasch-family and IRT diagnostics, response-category sensitivity analyses, construct-validity correlations, and exploratory abbreviated-form checks.

**Results:**

Parallel analysis supported one factor. The one-factor CFA showed strong comparative and residual fit, CFI = 0.999, TLI = 0.998, SRMR = 0.027, with RMSEA = 0.071. Loadings ranged from 0.823 to 0.874. Internal consistency was high, alpha = 0.94, 95% CI [0.928, 0.949], and omega total = 0.956, 95% CI [0.949, 0.966]. Partial credit item fit was acceptable, although lower thresholds were disordered; collapsing categories 2 and 3 restored ordered thresholds. CFQ-7 scores showed theoretically consistent associations with ACT-process, distress, self-evaluation, and vitality indicators. Exploratory four- and five-item variants closely tracked the full scale but require independent validation.

**Conclusion:**

Findings support the Swedish CFQ-7 as a reliable, predominantly unidimensional research measure, while indicating that response-category functioning, distress overlap, and replication require further study.

## Introduction

Cognitive fusion refers to relating to thoughts as literal, compelling events that can dominate behavior, rather than as ongoing verbal activity that can be noticed with distance. The Cognitive Fusion Questionnaire-7 (CFQ-7) was developed as a brief general measure of this process and is commonly used in acceptance and commitment therapy (ACT) and process-based research (Gillanders et al., 2014). Prior work supports the CFQ-7 as a useful measure across non-clinical and clinical psychological samples (Donati et al., 2021).

Swedish-language CFQ materials have been used in Swedish ACT and process research, including web-based ACT work where Swedish materials were translated and back-translated (Sairanen et al., 2019). However, Swedish item-level evidence has not been consolidated in a dedicated brief psychometric report. This creates a practical problem: the scale is already used, but evidence concerning internal structure, response-category functioning, and construct validity remains scattered.

The present brief report evaluates Swedish CFQ-7 item-level data from an adult Swedish-language sample. Because the data came from an existing broader dataset, the study is framed as an initial psychometric evaluation rather than as a definitive national validation or a direct test of translation equivalence. Consistent with the brief-report format, the focus is a targeted evaluation of item functioning, response-category performance, and construct-validity patterns for an already-used Swedish measure. Brevity is also substantively relevant: if a still shorter Swedish CFQ form can be supported in future work, it may reduce participant burden in intensive, clinical, or multi-measure process studies. The central question is whether Swedish CFQ-7 scores show sufficient reliability, practical unidimensionality, response-category coherence, and theoretically expected construct-validity associations to be useful in research.

## Materials and methods

### Participants and procedure

The study used secondary analyses of an existing Swedish general-population dataset. The dataset contained 432 adult respondents who completed the survey in Swedish. Complete CFQ-7 item data were available for 429 respondents (99.3%). Valid age data were available for 427 respondents; ages ranged from 18 to 82 years, with a mean age of approximately 48 years. The sample included 232 women, 199 men, and one respondent reporting another gender. The same dataset has also supported other manuscript work in the research program; the present analyses address a distinct psychometric question concerning the Swedish CFQ-7. The study was approved by the Swedish Ethical Review Authority (2022-07088-01).

### Measures

The CFQ-7 (Gillanders et al., 2014) contains seven items rated from 1 to 7, with higher scores indicating greater cognitive fusion. The Swedish item wording used here belongs to the same Swedish CFQ-7 lineage as prior Swedish-language ACT/process research, including Swedish applications that described translation/back-translation procedures or ACT-expert translation work (Sairanen et al., 2019). The present study did not directly compare Swedish wording variants or test linguistic equivalence against the original English items. Full item wording is not reproduced because of copyright and permissions considerations.

Construct validity was examined using theoretically relevant criterion variables available in the public analysis dataset. ACT/process indicators came from the 24-item Multidimensional Psychological Flexibility Inventory (MPFI-24; Rolffs et al., 2018): MPFI fusion, MPFI psychological inflexibility, and MPFI defusion. Current-sample alpha values were 0.86, 0.88, and 0.80, respectively. Secondary indicators included the Patient Health Questionnaire-4 (PHQ-4; Kroenke et al., 2009; *α* = 0.89), five Screening Tool for Psychological Distress indicators (STOP-D; Young et al., 2007), the Single-Item Self-Esteem Scale (SISE; Robins et al., 2001), and a three-item vitality score (*α* = 0.91). STOP-D and SISE indicators were single-item measures, so internal consistency was not applicable.

### Analyses

*Design and analysis transparency*: We report how the sample was obtained, all CFQ-7 variables analyzed for this study, all data exclusions, and all central model comparisons. Inclusion/exclusion criteria were not preregistered before data analysis. Data exclusions were limited to analysis-specific missing-data handling. CFQ-7 item-level analyses used complete CFQ-7 item data; criterion correlations used pairwise available data, which accounts for the small variation in n across external variables. No preregistered analysis plan was available for this secondary psychometric analysis.

Internal consistency was estimated using Cronbach’s alpha and omega total, with percentile bootstrap confidence intervals. Dimensionality was examined using parallel analysis and a one-factor exploratory factor analysis. A one-factor CFA was estimated using WLSMV with CFQ items treated as ordered categorical indicators. Fit was evaluated using CFI, TLI, RMSEA, and SRMR, interpreted jointly. As a sensitivity analysis, empirical dynamic fit-index cutoffs were applied to the one-factor ordinal CFA, with the lavaan fitted object set to use the scaled-shifted test option before cutoff estimation.

Rasch-family diagnostics used a partial credit model with item infit/outfit and category-threshold inspection. A graded response model was estimated as a complementary IRT model. Although both models belong to the IRT family, they served different purposes. The partial credit model was used to evaluate item fit and response-category threshold ordering under a Rasch-family equal-discrimination framework. The graded response model allowed item discriminations to vary and provided a less restrictive check of item-level fit and latent response patterns. Response-category sensitivity analyses compared the original seven-category scoring with adjacent-category collapse schemes. Construct validity was examined through Pearson correlations between CFQ total scores and theoretically relevant variables. Exploratory abbreviated-form checks were conducted as same-sample sensitivity analyses to identify promising item subsets for future work, not to validate a new short form.

Analyses were conducted in R 4.5.3 (R Core Team, 2026). Descriptives, parallel analysis, and reliability estimates used psych 2.6.3 (Revelle, 2026). Ordinal CFA models were estimated with lavaan 0.6–21 using WLSMV estimation (Rosseel, 2012). Partial credit model diagnostics used eRm 1.0–10 (Mair and Hatzinger, 2007), graded response modeling used mirt 1.46.1 (Chalmers, 2012), and empirical dynamic fit-index cutoffs used dynamic 1.1.0 (Wolf and McNeish, 2025). Data-wrangling scripts and derived outputs are included in the open-science package.

## Results

Of 432 respondents, 429 had complete CFQ-7 data. Item means ranged from 3.28 to 3.73 on the 1–7 scale, with standard deviations from 1.72 to 1.88, and all items used the full response range (Table 1). Internal consistency was high: alpha = 0.94, 95% CI [0.928, 0.949], and omega total = 0.956, 95% CI [0.949, 0.966]. Omega hierarchical was 0.682 and is reported descriptively because its bootstrap interval was unstable. Corrected item-total/drop correlations ranged from 0.762 to 0.828.

**Table 1:** Item descriptives for the Swedish CFQ-7.

Item	*M*	*SD*	Median
CFQ1	3.38	1.72	4
CFQ2	3.28	1.74	3
CFQ3	3.54	1.78	4
CFQ4	3.67	1.88	4
CFQ5	3.40	1.77	4
CFQ6	3.39	1.74	4
CFQ7	3.73	1.80	4

**Figure 1:**
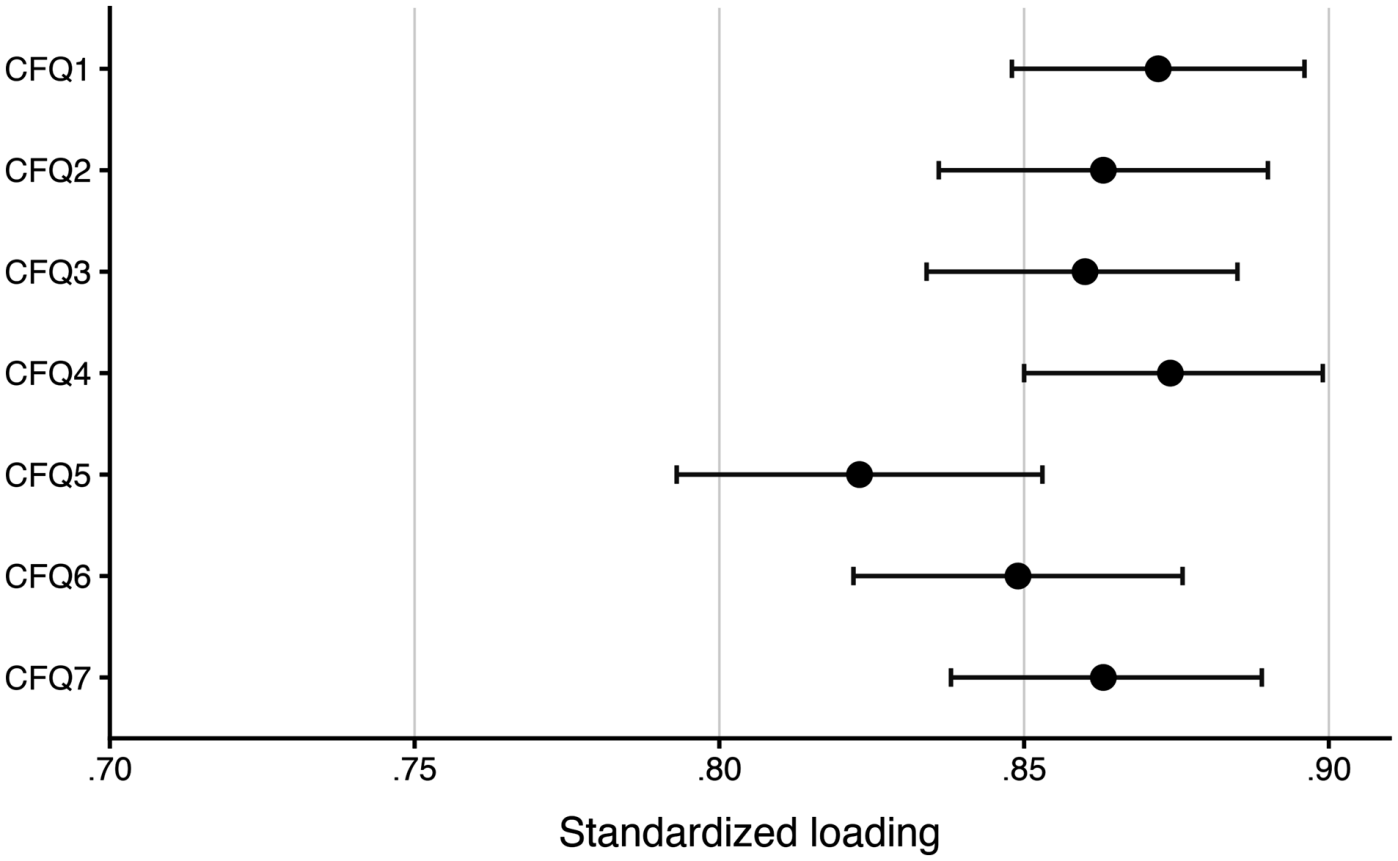
Standardized factor loadings for the one-factor model of the Swedish CFQ-7. Error bars show 95% confidence intervals. The model was estimated using WLSMV with CFQ items treated as ordered categorical indicators. CFQ = Cognitive Fusion Questionnaire.

Parallel analysis suggested one factor. In the one-factor CFA, model fit was strong on comparative and residual indices: CFI = 0.999, TLI = 0.998, SRMR = 0.027. RMSEA was 0.071, indicating moderate absolute misfit by conventional cutoffs. Standardized loadings were uniformly high, ranging from 0.823, 95% CI [0.793, 0.853], to 0.874, 95% CI [0.850, 0.899] (Figure 1).

Dynamic fit-index cutoffs provided a stricter empirical check. At the least restrictive available level, the one-factor model did not pass all dynamic criteria: SRMR = 0.027 exceeded the empirical cutoff of 0.021, scaled RMSEA = 0.153 exceeded the cutoff of 0.092, and scaled CFI = 0.986 met the cutoff of 0.981. Thus, dynamic fit evidence supported the one-factor model as useful and parsimonious, but not unequivocally well fitting under empirical absolute-fit expectations.

Rasch-family partial credit item fit was generally acceptable. Outfit mean squares ranged from 0.817 to 1.062, and infit mean squares ranged from 0.772 to 1.041. Several items showed negative standardized fit values, suggesting overfit rather than underfit. Threshold inspection showed systematic disordering between lower response thresholds corresponding to categories 2 and 3 across all items, suggesting that respondents may not sharply distinguish these adjacent lower response categories. Collapsing categories 2 and 3 was the least invasive recoding strategy that restored ordered thresholds for all seven items while preserving acceptable item fit.

A complementary graded response model showed acceptable comparative fit, CFI = 0.982 and TLI = 0.973, and residual fit, SRMSR = 0.050, but elevated RMSEA = 0.113. Item-level S-X2 tests were nonsignificant for six of seven items; CFQ7 showed statistically significant item misfit, *p* = 0.023.

CFQ total scores showed theoretically consistent construct-validity associations (Table 2). The strongest conceptually proximal associations were with ACT/process measures: MPFI fusion, *r* = 0.749 [0.704, 0.788], MPFI inflexibility, *r* = 0.732 [0.685, 0.773], and MPFI defusion, *r* = −0.278 [−0.363, −0.188]. CFQ-7 scores were also strongly associated with distress and stress indicators, including PHQ-4 mean, *r* = 0.766 [0.724, 0.803]. Wellbeing-related associations were in the expected direction, including negative correlations with self-esteem and vitality.

**Table 2:** Construct-validity correlations by interpretive domain.

Domain	Variable	*n*	*r*
ACT/process	MPFI fusion	429	0.749
ACT/process	MPFI inflexibility	429	0.732
ACT/process	MPFI defusion	428	−0.278
Distress/stress	PHQ-4 mean	429	0.766
Distress/stress	STOP-D sadness	424	0.668
Distress/stress	STOP-D anxiety	424	0.728
Distress/stress	STOP-D stress	424	0.699
Distress/stress	STOP-D anger	422	0.566
Distress/stress	STOP-D lack of social support	421	0.638
Wellbeing	SISE self-esteem	429	−0.399
Wellbeing	Vitality mean	426	−0.438

Exploratory abbreviated-form checks suggested that shorter item sets can approximate the full CFQ-7 total score in this sample. A four-item candidate comprising CFQ2, CFQ3, CFQ4, and CFQ6 showed alpha = 0.896, correlated 0.985 with the full CFQ-7 total score, and had strong conventional one-factor CFA fit, CFI = 1.000, TLI = 0.999, RMSEA = 0.040, and SRMR = 0.012; however, it did not pass all dynamic cutoff criteria because scaled RMSEA remained above the empirical cutoff. A five-item candidate comprising CFQ1, CFQ3, CFQ4, CFQ5, and CFQ7 showed alpha = 0.917, correlated 0.992 with the full CFQ-7, and passed all dynamic cutoff criteria at the Level-2 threshold. Both candidates retained strong associations with MPFI fusion and inflexibility, and their PHQ-4 correlations were close to the full CFQ-7 correlation. Because item selection and evaluation occurred in the same sample, these findings should be treated as hypothesis-generating.

## Discussion

The Swedish CFQ-7 showed strong internal consistency, low missingness, full use of the response range, high item-total associations, and uniformly high factor loadings. These findings support the CFQ-7 as a reliable and predominantly unidimensional measure of cognitive fusion in this Swedish-language adult sample.

The structural findings also warrant a cautious interpretation. Parallel analysis supported one factor, and the one-factor CFA showed excellent CFI, TLI, and SRMR. However, RMSEA was elevated and dynamic fit-index cutoffs did not support unequivocal absolute fit. This pattern suggests that the CFQ-7 is practically unidimensional for research scoring, while still showing residual psychometric complexity. In ACT terms, this is not surprising: items intended to capture behaviorally dominant thoughts are likely to sit close in content and function.

The Rasch-family findings point to response-category functioning as a specific issue. Item fit was broadly acceptable, but lower response thresholds were disordered across items. Collapsing categories 2 and 3 resolved the disordering in sensitivity analyses, indicating that the problem was localized to adjacent low-intensity distinctions rather than to item content as such. For comparability with prior CFQ-7 research, the original 1–7 scoring should remain the primary scoring approach, with collapsed-category results treated as supplementary sensitivity evidence.

The threshold finding is practically relevant because future Swedish CFQ-7 work should examine whether respondents distinguish adjacent low-intensity response options as intended, for example through cognitive interviewing or planned response-category studies. For now, the finding is better read as a warning about lower-end scale use than as a reason to abandon conventional scoring.

The exploratory four- and five-item findings are promising but preliminary. The four-item candidate may be attractive for brevity and content economy, whereas the five-item candidate showed the stronger dynamic-fit sensitivity pattern. These results suggest possible shorter Swedish CFQ item sets for future evaluation, especially where participant burden is a central design constraint, but the present study cannot establish a validated short form because item selection and evaluation occurred in the same dataset.

Construct-validity findings were theoretically coherent. CFQ-7 scores were strongly associated with MPFI fusion and broader psychological inflexibility, negatively associated with MPFI defusion, and negatively associated with self-esteem and vitality. At the same time, associations with distress and stress were also strong. This is plausible because when thoughts are responded to as literal and compelling, they are unlikely to be affectively neutral. Still, the strength of these associations means the CFQ-7 should not be treated as a process-pure measure independent of general negative affect.

Several limitations should be noted. The analyses used an existing dataset rather than an independent validation sample. The study did not include a formal translation-equivalence design, bilingual comparison, cognitive interviewing, test–retest reliability, measurement invariance, residual-correlation modeling, or response-process evidence. These are natural next steps given the strict-fit and dynamic-cutoff findings. National representativeness should not be assumed unless confirmed from recruitment documentation. The cross-sectional self-report design also means that criterion associations should be interpreted as preliminary construct-validity evidence rather than causal, diagnostic, treatment-sensitivity, or predictive evidence.

In summary, the Swedish CFQ-7 showed strong reliability, practical unidimensionality, broadly acceptable item fit, and theoretically consistent construct-validity correlations. The evidence supports the Swedish CFQ-7 as a useful research measure of cognitive fusion, while indicating that response-category functioning, distress overlap, measurement invariance, temporal stability, and independent replication warrant further study.

## Data Availability

The datasets presented in this study can be found in online repositories. The names of the repository/repositories and accession number(s) can be found at: https://osf.io/3h9zm.
